# Cancer prevalence and its determinants in Hungary: Analyzing data from the 2009, 2014, and 2019 European Health Interview Surveys

**DOI:** 10.1371/journal.pone.0315689

**Published:** 2025-02-13

**Authors:** Amr Sayed Ghanem, Eszter Vargáné Faludi, Róbert Bata, Emese Mezei, Vanessza Hadar, Marianna Móré, Ágnes Tóth, Attila Csaba Nagy

**Affiliations:** 1 Department of Health Informatics, Faculty of Health Sciences, University of Debrecen, Debrecen, Hungary; 2 Department of Integrative Health Sciences, Faculty of Health Sciences, University of Debrecen, Debrecen, Hungary; 3 Institute of Social and Sociological Sciences, Faculty of Health Sciences, University of Debrecen, Nyíregyháza, Hungary; Ruhr University Bochum, GERMANY

## Abstract

**Background and aim** Hungary has the fifth highest cancer incidence rate in the European Union, with an age-standardized rate (ASR) of 336.7 per 100,000 according to GLOBOCAN 2022. Additionally, Hungary holds the highest cancer mortality rate in the EU, with an ASR of 148.1 per 100,000. This study aimed to investigate the sociodemographic, lifestyle, and chronic disease-related factors affecting cancer prevalence in the Hungarian population. **Materials and methods** Data from the 2009, 2014, and 2019 installments of the European Health Interview Survey conducted in Hungary were pooled, resulting in a representative sample of 16,480 individuals. Weighted multiple logistic regression models were used to analyze the data, with goodness of fit assessed using the Hosmer-Lemeshow test. The best-fitting models were further evaluated using ROC analysis to calculate the Area Under the Curve (AUC) to assess discriminative ability. **Results** Urban residency was associated with higher cancer odds in 2014 (OR 1.85 [CI: 1.08–3.16]) and the pooled data (OR 1.44 [CI: 1.08–1.9]). Employed individuals had lower odds of cancer (2014: OR 0.34 [CI: 0.16–0.74]; pooled: OR 0.64 [CI: 0.45–0.92]). Among comorbid conditions, peptic ulcer (2009: OR 1.74 [CI: 1.13–2.69]; 2019: OR 3.2 [CI: 1.58–6.47]; pooled: OR 1.83 [CI: 1.31–2.54]) and chronic liver disease (2009: OR 3.52 [CI: 1.73–7.17]; pooled: OR 2.5 [CI: 1.4–4.47]) were significantly associated with higher cancer odds. Reporting bad health was linked to increased cancer risk (2009: OR 2.92 [CI: 1.87–4.58]; 2014: OR 5.52 [CI: 3.23–9.45]; 2019: OR 2.23 [CI: 1.26–3.95]). **Conclusion** Comorbid conditions such as peptic ulcer and chronic liver disease significantly increase cancer risk in Hungary. Urban residents require targeted preventive measures, and unemployment should be addressed. Early detection through appropriate screening and effective management of comorbid conditions are essential to prevent escalation and reduce overall cancer prevalence.

## 1 Introduction

Cancer is one of the leading causes of death worldwide, and in terms of premature death, it is considered the primary cause of death in some countries [1,2].

The global burden of cancer remains substantial; in 2022, GLOBOCAN estimated nearly 20 million new cases of cancer worldwide, indicating that approximately one in five men or women will develop cancer over their lifetime

[3]. According to some demographics-based data, the number of new cases will reach 35 million by 2050, that is, a 77% increase from the number in 2022 [4]. Parallel to the increase in the number of new cases, the burden caused by cancer death also elevates: from 2010 to 2019 these represented a 26.3% increase in new cases, a 20.9% increase in deaths, and a 16.0% increase in disability-adjusted life years (DALYs) [5]. Globally 9.7 million deaths can be linked to cancer according to the GLOBOCAN 2022; lung cancer was the leading cause of cancer death, with an estimated 1.8 million deaths (18.7%), followed by colorectal (9.3%), liver (7.8%), female breast (6.9%), and stomach (6.8%) cancers [3].

In Hungary, 30,456 people died due to cancer in 2022, with a standardized death rate of 143.7 per 100,000 population, according to GLOBOCAN estimates using the Segi-Doll standard [3]. This rate places Hungary among the highest in cancer mortality in Europe, accounting for 22.3% of total annual deaths [6]. Hungary has consistently reported some of the highest rates of lung cancer incidence and mortality worldwide [7].

The cause of cancer development is mainly external; environmental exposures are responsible for the 80–90% of cancers [8]. It is now almost self-evident that some of the lifestyle habits can contribute to the development of cancer as well; in 2019 the major risk factors were smoking, alcohol consumption and high body mass index (BMI) [9,10]. In addition to these, reproductive behaviour can also contribute to the development of certain types of malignant tumors [8]. However, there are other factors that can increase the risk of cancer, such as marital status, place of residence or educational level. Some types of cancer have higher incidence in metropolitan areas, while studies have described higher incidence rates for smoking-related cancers (e.g., lung and bladder cancer) and human papillomavirus (HPV)-related cancers (e.g., cervix cancer) in rural areas [11]. Educational level can also affect the risk of developing cancer, although the direction of the influence is not clear: for some tumor types, higher education appears as a risk factor, while for others it is on the contrary, a protective factor. The prevalence of some cancers (e.g. prostate, endometrial, melanoma and breast cancers) increases with increasing educational levels, whereas other cancer types (e.g. lung, gastric, esophageal, and nasopharyngeal cancers) are more frequent among people with low educational levels [12,13].

However, several studies have proven that the survival rate is more favourable for people with higher education. In terms of cancer mortality, American Cancer Society epidemiologists estimated that almost one-fourth (22%) of all cancer deaths would be preventable if all Americans had the cancer death rates of college-educated Americans [14]. This may also be due to the fact that according to some studies, people with higher education are more likely to attend regular screening tests, thereby contributing to an earlier diagnosis and the initiation of adequate treatment [15].

The main aim of the study was to determine the most important factors that can increase the likelihood of cancer; thus, the most vulnerable groups can be identified, which can be crucial in terms of developing more effective prevention strategies. To the authors’ knowledge, the current research is the first regionally to assess determinants of cancer prevalence using a large, nationally representative dataset, and a wide range of sociodemographic, lifestyle and health related determinants.

## 2 Materials and methods

### 2.1 Source of data

The European Health Interview Surveys (EHIS), conducted in 2009, 2014 and 2019 are an essential source of health indicators for the Hungarian population aged 15 and older living in private households. This repeated cross-sectional study employed a rigorous stratified sampling method to accurately reflect the health status of the population [16]. Data collection was performed both electronically and via direct interviews by using a standardized questionnaire across EU member states to maintain consistency and comparability. The data utilised in the study includes all the three iterations of the data collection related to the Hungarian population, which allows the monitoring of trends and the interrelatedness of health influencing factors between the individual years or when merging data to access cumulative health outcomes over the decade. As a result of merging the data deriving from the surveys conducted in 2009 (n = 5051), 2014 (n = 5826) and 2019 (n = 5603), the total number of responses adds up to 16480.

The research was performed in accordance with the principles of the Declaration of Helsinki and approved by the Regional Ethical Committee of University of Debrecen [5609-2020]. The database was accessed between the 5^th^ of January and 27^th^ of February 2024. All data used in this study were fully anonymized before access.

### 2.2 Range of examined variables

The underlying aim of the study is to reveal correlations with cancer as the primary outcome. Various independent variables were included to potentially indicate the presence and characteristics of the disease. The analysed variables incorporated socio-demographic factors such as gender (male, female), age groups (15–34, 35–64, 65+), relationships status (single, in relationship), education levels (lower than high school, high school, university), employment status (employed, unemployed), area of residence (rural, urban),

region of residence was classified according to the NUTS2 (Nomenclature of Territorial Units for Statistics, Level 2) categorization, which divides Hungary into seven regions: Central Hungary, Southern Great Plain, Southern Transdanubia, Northern Great Plain, Central Transdanubia, Northern Hungary, and Western Transdanubia. Within this framework, Central Hungary includes both Pest county and Budapest as a combined region.

Financial and income factors were considered as the measures of economic well-being. Financial status was expressed on a three-level scale including bad, average, and good categories, while income levels were divided into five tiers ranging from low to high. The available lifestyle factors are the following: body mass index (BMI) was classified as normal (BMI < 25) or overweight/obese (BMI  ≥  25); Smoking and alcohol use were categorized simply as engaging (smoker, drinker) or not engaging (non-smoker, non-drinker) in these behaviours. Self-perceived health was reported on a three-tier ordinal scale with good, average, and bad category labels. A wide range of medical conditions were examined in relation to cancer like asthma, bronchitis, cardiovascular diseases, hypertension, stroke, arthrosis, chronic back pain, chronic neck pain, diabetes, peptic ulcer, chronic liver disease, migraine, depression, mental illness, hypercholesterolaemia, arrhythmia. All of these conditions were represented with binary (yes, no) outcomes, standing for the presence or the absence of a condition.

### 2.3 Methods and statistical analysis

Weighted Pearson’s chi-squared tests were conducted to identify initial associations between variables and cancer occurrence, displaying simple correlations and weighted proportions. To account for confounding factors and assess independent effects, we further applied a weighted multiple logistic regression model, allowing for a more robust evaluation of variable associations with cancer occurrence.

Various diagnostic tools were employed to validate the fit and accuracy of the model, which included goodness-of-fit assessments using the Hosmer-Lemeshow test and the evaluation of model fit using the Akaike and Bayesian information criteria. These tools aided the selection of the most appropriate model for each outcome. To evaluate the predictive performance of the multiple logistic regression models, a Receiver Operating Characteristic (ROC) curve analysis was conducted. The ROC curve plots the true positive rate (sensitivity) against the false positive rate (1-specificity) at various threshold settings. The Area Under the Curve (AUC) was calculated to quantify the model's overall ability to discriminate between outcome groups, with values ranging from 0.5 (no discrimination) to 1.0 (perfect discrimination). This analysis was essential for assessing the model's discriminative power, providing a measure of performance beyond traditional accuracy metrics. The significance threshold was set at a p-value below 0.05, with results presented as adjusted odds ratios and with 95% confidence intervals, conducted using STATA IC Version 17.0.

## 3 Results

### 3.1 Key features of the 2009, 2014 and 2019 data

Results in Tables 1 and 2. indicated that the prevalence of cancer was significantly higher among women in *2009* (66,49%; p < 0.001). Participants aged 65 and above are the most heavily affected age group compared to the other groups (p < 0.001). Being unemployed is significantly associated with higher occurrence of cancer cases (78.68%; p < 0.001). Financial status implies strong link to cancer prevalence among those with bad financial status (p = 0.0079). Alcohol use was closely correlated to cancer occurrence (p < 0.001), with increased frequency among non-drinkers. Worse self-perceived health indicates higher prevalence of cancer (48.69%; p < 0.001). Taking prescribed medication highly influenced the prevalence of cancer (86,55%; p < 0.001). On the basis of the observed results, there is a wide array of chronic condition significantly associated with cancer (p < 0.001) like bronchitis, AMI (acute myocardial infarction), CAD (coronary artery disease), hypertension, arthrosis, back pain, neck pain, peptic ulcer, chronic liver disease, migraine, depression, mental illness, hypercholesterolaemia, arrhythmia.

In *2014* the 65 and above age group remained the most severely impacted age group (55.81%; p < 0.001). Cancer frequency remained steadily higher among the unemployed responders with a high association (p < 0.001). Regarding alcohol use, the occurence of cancer was higher among non-drinkers compared to drinkers (p < 0.001). Bad self-perceived health resulted in higher prevalence of cancer (58.29; p < 0.001). The following chronic conditions showed significant association with cancer (p < 0.001): AMI, CAD, hypertension, stroke, arthrosis, back pain, neck pain, peptic ulcer, depression arrythmia.

In *2019* age still represents a profound correlation to cancer, particularly the 65 and above age group were the most affected (53.61%; p < 0.001). The unemployed were more intensely associated to higher occurrence of cancer (67.93%; p < 0.001). Bad financial status is closely correlated with higher occurrence of cancer cases (p < 0.001). Cancer occurrence is significantly higher among non-drinkers compared to drinkers (p = 0.0012). Bad self-perceived health was more closely related to higher prevalence of cancer (p < 0.001). Taking prescription drug increased the frequency of cancer (88.36%; p < 0.001). The chronic conditions strongly linked to cancer prevalence (p < 0.001) are hypertension, stroke, arthrosis, neck pain diabetes, peptic ulcer, and arrhythmia.

### 3.2 Characteristics of the pooled dataset

The prevalence of cancer varied significantly within the cancer-affected group across the years surveyed: 2009, 2014, and 2019 as displayed in Tables 1 and 2. In 2009, the distribution of cancer cases was 51%, more than double that of 2014 (24.27%) and 2019 (24.66%), indicating a declining trend over the years. These differences reflect separate cross-sectional snapshots rather than a continuous longitudinal trend. There is a significant association between gender and cancer prevalence, females are having higher percentage of cancer cases to males (64.71% vs. 35.29%; p < 0.001). The 65 and above age groups shows a significantly higher percentage of cancer cases (49.54%) than the rest of the age groups. There is a notable difference in the prevalence of cancer between the employed and the unemployed responders, where the unemployed are more strongly affected with cancer cases (77.85%; p < 0.001). Financial status is significant in terms of the prevalence of cancer cases, ‘bad’ financial status is strongly correlated with higher rate of cancer (p < 0.001). Both smoking and alcohol use are significantly associated with cancer. Non-smokers and non-drinkers have higher prevalence of cancer cases in the studied population. Those respondents reporting ‘bad’ health are significantly more likely to have cancer (46.81%; p < 0.001). In the pooled sample all the chronic conditions included in the study showed significant correlation to the high incidence of cancer cases (p < 0.001). Each chronic condition like asthma, bronchitis, AMI, CAD, hypertension, stroke, arthrosis, diabetes, peptic ulcer, chronic liver disease, migraine, depression, mental illness, hypercholesterolemia, and arrhythmia, is associated with a significantly higher percentage of cancer cases among those afflicted compared to those without these conditions. Taking prescription drugs is strongly connected to the high occurrence of cancer cases (88.38%, p < 0.001). Those respondents who had undergone stool analysis have a higher proportion of cancer cases compared to those who did not.

**Table 1:** Sociodemographic and lifestlye characteristics of participants with cancer versus those without in the 2009, 2014, 2019 and pooled datasets with unweighted numbers and weighted proportions.

Characteristics		Cancer 2009 (n=179)				Cancer 2014 (n=102)				Cancer 2019 (n=118)				Cancer pooled (n=399)			
		not ill *(n, %)*	ill *(n, %)*	total *(n)*	p values	not ill *(n, %)*	ill *(n, %)*	total *(n)*	p values	not ill *(n, %)*	ill *(n, %)*	total *(n)*	p values	not ill *(n, %)*	ill *(n, %)*	total *(n)*	p values
**Year**														4870 (33.48)	179 (51.07)	5049 (33.9)	**<0.001**
														5722 (33.63)	102 (24.27)	5824 (33.4)	
														5421 (32.89)	118 (24.66)	5539 (32.7)	
**Gender**	Male	2239 (47.13)	58 (33.51)	2297 (46.64)	**<0.001**	2663 (47.13)	37 (36.12)	2700 (46.94)	**0.0324**	2493 (47.33)	45 (38.15)	2538 (47.17)	0.0627	7395 (47.2)	140 (35.29)	7535 (46.91)	**<0.001**
	Female	2631 (52.87)	121 (66.49)	2752 (53.36)		3059 (52.87)	65 (63.88)	3124 (53.06)		2928 (52.67)	73 (61.85)	3001 (52.83)		8618 (52.8)	259 (64.71)	8877 (53.09)	
**Age groups**	65+	969 (19.38)	77 (44.59)	1046 (20.29)	**<0.001**	1159 (20.03)	56 (55.81)	1215 (20.65)	**<0.001**	1541 (22.52)	68 (53.61)	1609 (23.08)	**<0.001**	3669 (20.63)	201 (49.54)	3870 (21.32)	**<0.001**
	15-34	1460 (31.58)	12 (8.65)	1472 (30.76)		1705 (28.91)	2 (1.63)	1707 (28.44)		1263 (27.61)	2 (2.53)	1265 (27.16)		4428 (29.38)	16 (5.44)	4444 (28.81)	
	35-64	2441 (49.03)	90 (46.76)	2531 (48.95)		2858 (51.06)	44 (42.56)	2902 (50.91)		2617 (49.88)	48 (43.86)	2665 (49.77)		7916 (49.99)	182 (45.02)	8098 (49.87)	
**Relationship status**	Single	2355 (46.61)	76 (40.41)	2431 (46.39)	0.1184	3019 (52.22)	43 (41.92)	3062 (52.04)	**0.0438**	2254 (41.38)	55 (46.25)	2309 (41.46)	0.3165	7628 (46.82)	174 (42.2)	7802 (46.71)	0.0834
	In relationship	2509 (53.39)	103 (59.59)	2612 (53.61)		2699 (47.78)	59 (58.08)	2758 (47.96)		3043 (58.62)	62 (53.75)	3105 (58.54)		8251 (53.18)	224 (57.8)	8475 (53.29)	
**Educational levels**	Lower than high school	2664 (53.22)	101 (55.33)	2765 (53.29)	0.8412	2923 (49.71)	50 (51)	2973 (49.73)	0.9465	2437 (42.25)	52 (43.26)	2489 (42.27)	0.959	8024 (48.43)	203 (51.29)	8227 (48.5)	0.5623
	High school	1491 (31.22)	51 (29.1)	1542 (31.14)		1713 (30.24)	30 (28.72)	1743 (30.21)		1783 (33.74)	38 (32.4)	1821 (33.71)		4987 (31.72)	119 (29.82)	5106 (31.67)	
	University	711 (15.56)	26 (15.56)	737 (15.56)		1085 (20.05)	22 (20.29)	1107 (20.05)		1201 (24.01)	28 (24.34)	1229 (24.02)		2997 (19.85)	76 (18.88)	3073 (19.83)	
**Employment**	Unemployed	2626 (52.61)	142 (78.68)	2768 (53.55)	**<0.001**	2858 (48.96)	86 (86.18)	2944 (49.61)	**<0.001**	2686 (44.84)	85 (67.93)	2771 (45.26)	**<0.001**	8170 (48.83)	313 (77.85)	8483 (49.52)	**<0.001**
	Employed	2244 (47.39)	37 (21.32)	2281 (46.45)		2858 (51.04)	16 (13.82)	2874 (50.39)		2735 (55.16)	33 (32.07)	2768 (54.74)		7837 (51.17)	86 (22.15)	7923 (50.48)	
**Residence area**	Rural	1427 (26.4)	50 (26.34)	1477 (26.4)	0.9877	1771 (28.86)	22 (20.28)	1793 (28.71)	0.0631	1769 (29.84)	27 (22.69)	1796 (29.71)	0.1061	4967 (28.36)	99 (23.97)	5066 (28.25)	0.0642
	Urban	3443 (73.6)	129 (73.66)	3572 (73.6)		3951 (71.14)	80 (79.72)	4031 (71.29)		3652 (70.16)	91 (77.31)	3743 (70.29)		11046 (71.64)	300 (76.03)	11346 (71.75)	
**Region**	Central-Hungary	1193 (29.73)	39 (25.37)	1232 (29.57)	0.1372	1460 (30.06)	29 (30.56)	1489 (30.07)	0.5916	1532 (30.64)	42 (37)	1574 (30.76)	0.4001	4185 (30.14)	110 (29.5)	4295 (30.13)	**0.0398**
	Southern Great Plain	697 (13.14)	31 (15.97)	728 (13.24)		799 (13.02)	11 (10.69)	810 (12.97)		675 (12.87)	13 (11.35)	688 (12.84)		2171 (13.01)	55 (13.55)	2226 (13.02)	
	Southern Transdanubia	474 (9.33)	26 (14.67)	500 (9.52)		558 (9.24)	13 (13.3)	571 (9.31)		514 (9.05)	13 (10.23)	527 (9.07)		1546 (9.21)	52 (13.24)	1598 (9.3)	
	Northern Great Plain	797 (14.77)	23 (11.46)	820 (14.65)		966 (14.88)	13 (11.62)	979 (14.82)		856 (14.88)	9 (7.21)	865 (14.74)		2619 (14.84)	45 (10.45)	2664 (14.74)	
	Central Transdanubia	532 (10.86)	23 (13.3)	555 (10.95)		665 (10.85)	16 (14.58)	681 (10.92)		600 (10.94)	13 (10.97)	613 (10.94)		1797 (10.89)	52 (13.04)	1849 (10.94)	
	Northern Hungary	650 (12.03)	22 (11.25)	672 (12)		684 (11.78)	12 (11.52)	696 (11.78)		680 (11.4)	13 (11.4)	693 (11.4)		2014 (11.74)	47 (11.35)	2061 (11.73)	
	Western Transdanubia	527 (10.15)	15 (7.97)	542 (10.07)		590 (10.17)	8 (7.73)	598 (10.13)		564 (10.22)	15 (11.84)	579 (10.25)		1681 (10.18)	38 (8.87)	1719 (10.15)	
**Financial status**	Average	2819 (58.71)	91 (50.82)	2910 (58.42)	**0.0079**	3259 (56.93)	59 (59.6)	3318 (56.98)	0.1382	3041 (55.93)	63 (52.51)	3104 (55.87)	**<0.001**	9119 (57.2)	213 (53.37)	9332 (57.11)	**<0.001**
	Good	617 (13.05)	16 (9.66)	633 (12.93)		1211 (21.78)	15 (14.05)	1226 (21.65)		1639 (32.81)	24 (21.65)	1663 (32.6)		3467 (22.43)	55 (13.65)	3522 (22.22)	
	Bad	1396 (28.24)	71 (39.52)	1467 (28.65)		1206 (21.29)	27 (26.35)	1233 (21.38)		626 (11.26)	28 (25.84)	654 (11.52)		3228 (20.36)	126 (32.98)	3354 (20.66)	
**Income quintiles**	First	966 (19.94)	37 (21.45)	1003 (20)	0.2828	1137 (19.37)	28 (25.12)	1165 (19.47)	0.0669	1125 (20.2)	20 (16.62)	1145 (20.14)	**0.0303**	3228 (19.84)	85 (21.15)	3313 (19.87)	0.3648
	Second	965 (20.2)	28 (15.28)	993 (20.02)		1143 (19.32)	21 (22.16)	1164 (19.37)		1127 (19.85)	36 (29.94)	1163 (20.03)		3235 (19.79)	85 (20.56)	3320 (19.81)	
	Third	953 (19.96)	36 (20.44)	989 (19.98)		1230 (21.15)	26 (27.08)	1256 (21.25)		1099 (19.93)	28 (24.66)	1127 (20.01)		3282 (20.35)	90 (23.09)	3372 (20.42)	
	Fourth	1005 (19.9)	47 (25.48)	1052 (20.1)		1043 (18.59)	11 (10.7)	1054 (18.46)		1233 (22.62)	19 (14.51)	1252 (22.47)		3281 (20.35)	77 (19.19)	3358 (20.33)	
	Fifth	981 (19.99)	31 (17.35)	1012 (19.9)		1169 (21.57)	16 (14.95)	1185 (21.45)		837 (17.4)	15 (14.27)	852 (17.35)		2987 (19.67)	62 (16.01)	3049 (19.58)	
**BMI**	Overweight/Obese	2635 (53.51)	106 (59.3)	2741 (53.72)	0.1467	3065 (53.84)	61 (60.71)	3126 (53.96)	0.1812	3215 (57.9)	77 (65.61)	3292 (58.04)	0.1124	8915 (55.06)	244 (61.19)	9159 (55.21)	**0.0216**
	Normal	2222 (46.49)	73 (40.7)	2295 (46.28)		2634 (46.16)	40 (39.29)	2674 (46.04)		2151 (42.1)	40 (34.39)	2191 (41.96)		7007 (44.94)	153 (38.81)	7160 (44.79)	
**Smoking**	Smoker	1480 (31.66)	43 (25.16)	1523 (31.43)	0.0839	1590 (27.63)	20 (20.71)	1610 (27.51)	0.1361	1491 (28.07)	25 (19.45)	1516 (27.91)	**0.0401**	4561 (29.1)	88 (22.58)	4649 (28.95)	**0.0071**
	Non-smoker	3263 (68.34)	124 (74.84)	3387 (68.57)		4124 (72.37)	82 (79.29)	4206 (72.49)		3930 (71.93)	93 (80.55)	4023 (72.09)		11317 (70.9)	299 (77.42)	11616 (71.05)	
**Alcohol use**	Drinker	2993 (63.56)	83 (48.27)	3076 (63.01)	**<0.001**	3971 (70.48)	54 (52.08)	4025 (70.16)	**<0.001**	3787 (71.4)	67 (57.05)	3854 (71.14)	**0.0012**	10751 (68.49)	204 (51.38)	10955 (68.08)	**<0.001**
	Non-drinker	1818 (36.44)	94 (51.73)	1912 (36.99)		1738 (29.52)	48 (47.92)	1786 (29.84)		1634 (28.6)	51 (42.95)	1685 (28.86)		5190 (31.51)	193 (48.62)	5383 (31.92)	
**Self-perceived health**	Average	1549 (31.11)	58 (32.75)	1607 (31.16)	**<0.001**	1587 (27.52)	30 (29.03)	1617 (27.55)	**<0.001**	1683 (28.31)	55 (48.38)	1738 (28.67)	**<0.001**	4819 (28.98)	143 (35.65)	4962 (29.14)	**<0.001**
	Good	2636 (55.53)	30 (18.56)	2666 (54.2)		3533 (62.26)	13 (12.67)	3546 (61.4)		3145 (62.44)	20 (20.26)	3165 (61.69)		9314 (60.06)	63 (17.54)	9377 (59.05)	
	Bad	684 (13.36)	91 (48.69)	775 (14.63)		602 (10.22)	59 (58.29)	661 (11.05)		574 (9.24)	41 (31.36)	615 (9.64)		1860 (10.95)	191 (46.81)	2051 (11.81)	

Bold values indicate statistical significance, p<0.05 based on weighted Pearson’s chi squared test

**Table 2:** Chronic disease related characteristics of participants according to the 2009, 2014, 2019 and pooled datasets.

Characteristics		Cancer 2009 (n = 179)				Cancer 2014 (n = 102)				Cancer 2019 (n = 118)				Cancer pooled (n = 399)			
		not ill *(n, %)*	ill *(n, %)*	total *(n)*	p values	not ill *(n, %)*	ill *(n, %)*	total *(n)*	p values	not ill *(n, %)*	ill *(n, %)*	total *(n)*	p values	not ill *(n, %)*	ill *(n, %)*	total *(n)*	p values
**Asthma**	No	4563 (93.62)	160 (90.18)	4723 (93.49)	0.0675	5432 (95.18)	92 (90.17)	5524 (95.09)	**0.0235**	5129 (95.16)	109 (92.16)	5238 (95.1)	0.1777	15124 (94.65)	361 (90.66)	15485 (94.55)	**<0.001**
	Yes	307 (6.38)	19 (9.82)	326 (6.51)		289 (4.82)	10 (9.83)	299 (4.91)		276 (4.84)	8 (7.84)	284 (4.9)		872 (5.35)	37 (9.34)	909 (5.45)	
**Bronchitis**	No	4575 (93.98)	149 (84.18)	4724 (93.63)	**<0.001**	5485 (95.94)	92 (90.99)	5577 (95.85)	**0.0158**	5167 (95.98)	107 (91.17)	5274 (95.89)	**0.013**	15227 (95.3)	348 (87.56)	15575 (95.11)	**<0.001**
	Yes	295 (6.02)	29 (15.82)	324 (6.37)		237 (4.06)	9 (9.01)	246 (4.15)		232 (4.02)	11 (8.83)	243 (4.11)		764 (4.7)	49 (12.44)	813 (4.89)	
**AMI**	No	4671 (95.94)	162 (90.76)	4833 (95.75)	**<0.001**	5587 (97.75)	95 (92.88)	5682 (97.66)	**<0.001**	5266 (97.84)	112 (96.79)	5378 (97.82)	0.3897	15524 (97.17)	369 (92.76)	15893 (97.07)	**<0.001**
	Yes	199 (4.06)	17 (9.24)	216 (4.25)		135 (2.25)	7 (7.12)	142 (2.34)		142 (2.16)	5 (3.21)	147 (2.18)		476 (2.83)	29 (7.24)	505 (2.93)	
**CAD**	No	4524 (93.17)	147 (81.72)	4671 (92.76)	**<0.001**	5432 (95.29)	84 (83.12)	5516 (95.08)	**<0.001**	5164 (96.45)	110 (94.16)	5274 (96.41)	0.2046	15120 (94.96)	341 (85.13)	15461 (94.73)	**<0.001**
	Yes	345 (6.83)	32 (18.28)	377 (7.24)		289 (4.71)	18 (16.88)	307 (4.92)		233 (3.55)	8 (5.84)	241 (3.59)		867 (5.04)	58 (14.87)	925 (5.27)	
**Hypertension**	No	3267 (68.3)	78 (44.28)	3345 (67.43)	**<0.001**	3898 (68.52)	45 (42.89)	3943 (68.08)	**<0.001**	3515 (69.1)	47 (41.15)	3562 (68.6)	**<0.001**	10680 (68.64)	170 (43.18)	10850 (68.03)	**<0.001**
	Yes	1601 (31.7)	101 (55.72)	1702 (32.57)		1824 (31.48)	57 (57.11)	1881 (31.92)		1878 (30.9)	70 (58.85)	1948 (31.4)		5303 (31.36)	228 (56.82)	5531 (31.97)	
**Stroke**	No	4734 (97.27)	172 (96.16)	4906 (97.23)	0.3748	5591 (97.74)	93 (89.86)	5684 (97.6)	**<0.001**	5284 (98.07)	108 (93.12)	5392 (97.98)	**<0.001**	15609 (97.69)	373 (93.88)	15982 (97.6)	**<0.001**
	Yes	136 (2.73)	7 (3.84)	143 (2.77)		131 (2.26)	9 (10.14)	140 (2.4)		121 (1.93)	9 (6.88)	130 (2.02)		388 (2.31)	25 (6.12)	413 (2.4)	
**Arthrosis**	No	3736 (77.27)	94 (53.12)	3830 (76.41)	**<0.001**	4587 (80.52)	51 (48.77)	4638 (79.97)	**<0.001**	4304 (82.49)	67 (60.34)	4371 (82.1)	**<0.001**	12627 (80.07)	212 (53.79)	12839 (79.45)	**<0.001**
	Yes	1133 (22.73)	84 (46.88)	1217 (23.59)		1131 (19.48)	51 (51.23)	1182 (20.03)		1070 (17.51)	47 (39.66)	1117 (17.9)		3334 (19.93)	182 (46.21)	3516 (20.55)	
**Chronic back pain**	No	3335 (69.33)	94 (53.49)	3429 (68.76)	**<0.001**	3990 (69.99)	52 (48.97)	4042 (69.62)	**<0.001**	3661 (69.14)	63 (57.2)	3724 (68.93)	**0.0087**	10986 (69.49)	209 (53.28)	11195 (69.11)	**<0.001**
	Yes	1535 (30.67)	85 (46.51)	1620 (31.24)		1732 (30.01)	50 (51.03)	1782 (30.38)		1752 (30.86)	53 (42.8)	1805 (31.07)		5019 (30.51)	188 (46.72)	5207 (30.89)	
**Chronic neck pain**	No	4019 (83.29)	114 (64.51)	4133 (82.62)	**<0.001**	4903 (86.01)	70 (68.15)	4973 (85.7)	**<0.001**	4693 (87.71)	88 (75.64)	4781 (87.5)	**<0.001**	13615 (85.66)	272 (68.1)	13887 (85.24)	**<0.001**
	Yes	850 (16.71)	65 (35.49)	915 (17.38)		819 (13.99)	32 (31.85)	851 (14.3)		718 (12.29)	28 (24.36)	746 (12.5)		2387 (14.34)	125 (31.9)	2512 (14.76)	
**Diabetes**	No	4468 (91.87)	155 (86.32)	4623 (91.67)	**0.0119**	5262 (92.07)	86 (84.44)	5348 (91.94)	**0.0064**	4858 (91.35)	98 (81.21)	4956 (91.17)	**<0.001**	14588 (91.77)	339 (84.6)	14927 (91.6)	**<0.001**
	Yes	402 (8.13)	24 (13.68)	426 (8.33)		459 (7.93)	16 (15.56)	475 (8.06)		524 (8.65)	20 (18.79)	544 (8.83)		1385 (8.23)	60 (15.4)	1445 (8.4)	
**Peptic ulcer**	No	4489 (92.35)	132 (76.21)	4621 (91.77)	**<0.001**	5529 (96.63)	90 (88.73)	5619 (96.49)	**<0.001**	5256 (97.44)	101 (86.84)	5357 (97.25)	**<0.001**	15274 (95.46)	323 (81.86)	15597 (95.14)	**<0.001**
	Yes	381 (7.65)	47 (23.79)	428 (8.23)		191 (3.37)	12 (11.27)	203 (3.51)		148 (2.56)	16 (13.16)	164 (2.75)		720 (4.54)	75 (18.14)	795 (4.86)	
**Chronic liver disease**	No	4820 (99.02)	165 (92.87)	4985 (98.8)	**<0.001**	5693 (99.53)	99 (97.99)	5792 (99.5)	**0.0109**	5373 (99.45)	115 (99.52)	5488 (99.45)	0.896	15886 (99.33)	379 (95.74)	16265 (99.25)	**<0.001**
	Yes	50 (0.98)	14 (7.13)	64 (1.2)		28 (0.47)	3 (2.01)	31 (0.5)		31 (0.55)	1 (0.48)	32 (0.55)		109 (0.67)	18 (4.26)	127 (0.75)	
**Migraine**	No	4048 (83.2)	114 (63.9)	4162 (82.51)	**<0.001**	5011 (87.86)	83 (81.29)	5094 (87.75)	0.0536	4777 (87.46)	109 (92.99)	4886 (87.56)	0.0772	13836 (86.17)	306 (75.29)	14142 (85.91)	**<0.001**
	Yes	822 (16.8)	65 (36.1)	887 (17.49)		711 (12.14)	19 (18.71)	730 (12.25)		636 (12.54)	9 (7.01)	645 (12.44)		2169 (13.83)	93 (24.71)	2262 (14.09)	
**Depression**	No	4574 (94.32)	143 (80.12)	4717 (93.81)	**<0.001**	5442 (95.21)	89 (87.99)	5531 (95.09)	**<0.001**	5164 (96.07)	107 (92.15)	5271 (96)	**0.0497**	15180 (95.2)	339 (84.97)	15519 (94.95)	**<0.001**
	Yes	296 (5.68)	36 (19.88)	332 (6.19)		280 (4.79)	13 (12.01)	293 (4.91)		216 (3.93)	9 (7.85)	225 (4)		792 (4.8)	58 (15.03)	850 (5.05)	
**Mental illnesses**	No	4708 (96.78)	163 (92.26)	4871 (96.62)	**<0.001**	5548 (97.08)	93 (91.96)	5641 (96.99)	**0.0023**	4887 (91.33)	95 (83.54)	4982 (91.2)	**0.0059**	15143 (95.11)	351 (90.09)	15494 (94.99)	**<0.001**
	Yes	162 (3.22)	16 (7.74)	178 (3.38)		173 (2.92)	9 (8.04)	182 (3.01)		472 (8.67)	19 (16.46)	491 (8.8)		807 (4.89)	44 (9.91)	851 (5.01)	
**Hypercholesterolaemia**	No	4272 (88.13)	128 (70.51)	4400 (87.5)	**<0.001**	5075 (88.97)	83 (82.66)	5158 (88.86)	**0.0435**	4568 (87.08)	88 (78.66)	4656 (86.93)	**0.0109**	13915 (88.07)	299 (75.43)	14214 (87.77)	**<0.001**
	Yes	589 (11.87)	51 (29.49)	640 (12.5)		633 (11.03)	19 (17.34)	652 (11.14)		743 (12.92)	25 (21.34)	768 (13.07)		1965 (11.93)	95 (24.57)	2060 (12.23)	
**Arrhythmia**	No	4194 (86.46)	118 (65.78)	4312 (85.72)	**<0.001**	5150 (90.43)	67 (66.28)	5217 (90.01)	**<0.001**	4820 (90.76)	93 (79.74)	4913 (90.56)	**<0.001**	14164 (89.2)	278 (69.31)	14442 (88.73)	**<0.001**
	Yes	675 (13.54)	61 (34.22)	736 (14.28)		571 (9.57)	35 (33.72)	606 (9.99)		561 (9.24)	24 (20.26)	585 (9.44)		1807 (10.8)	120 (30.69)	1927 (11.27)	
**Taking prescripti on drugs**	No	2308 (48.53)	22 (13.45)	2330 (47.27)	**<0.001**	2905 (50.94)	8 (7.76)	2913 (50.19)	**<0.001**	2471 (49.8)	14 (11.64)	2485 (49.11)	**<0.001**	7684 (49.76)	44 (11.62)	7728 (48.85)	**<0.001**
	Yes	2561 (51.47)	157 (86.55)	2718 (52.73)		2816 (49.06)	94 (92.24)	2910 (49.81)		2928 (50.2)	104 (88.36)	3032 (50.89)		8305 (50.24)	355 (88.38)	8660 (51.15)	
**Taking OTC drugs or supplements**	No	3131 (63.53)	87 (48.84)	3218 (63)	**<0.001**	3065 (52.78)	48 (48.63)	3113 (52.71)	0.4171	1179 (20.07)	23 (20.99)	1202 (20.08)	0.8197	7375 (45.65)	158 (41.92)	7533 (45.56)	0.1647
	Yes	1737 (36.47)	92 (51.16)	1829 (37)		2656 (47.22)	54 (51.37)	2710 (47.29)		4220 (79.93)	95 (79.01)	4315 (79.92)		8613 (54.35)	241 (58.08)	8854 (54.44)	
**Stool analysis**	No	275 (65.82)	34 (66.15)	309 (65.85)	0.9635	5094 (89.48)	76 (75.02)	5170 (89.23)	**<0.001**	1048 (66.88)	31 (48.12)	1079 (66.24)	**0.0034**	6417 (83.39)	141 (65.59)	6558 (82.91)	**<0.001**
	Yes	139 (34.18)	18 (33.85)	157 (34.15)		602 (10.52)	25 (24.98)	627 (10.77)		528 (33.12)	33 (51.88)	561 (33.76)		1269 (16.61)	76 (34.41)	1345 (17.09)	

Bold values indicate statistical significance, p < 0.05 based on weighted Pearson’s chi squared test

### 3.3 Results of multiple logistic regression models in 2009, 2014 and 2019 data

As displayed in Table 3, in *2009* the younger age groups, 15–34 (OR: 0.26 [CI: 0.12–0.58]) and 35–64 age groups (0.54 [0.34–0.85]), indicated significantly lower odds of having cancer compared to those aged 65 or above. Having higher than lower-level education increased the odds of cancer, particularly with university level education (2.6 [1.49–4.55]). Reporting bad health was strongly linked to increased risk of having cancer (2.92 [1.87–4.58]), but reporting good health was linked to lower risk (0.49 [0.28–0.85]). There were several chronic conditions associated with decreased odds of cancer like stroke (0.42 [0.18–0.99]) and back pain (0.65 [0.43–0.99]). However, some conditions were closely associated with the increase in the odds including peptic ulcer (1,74 [1.13–2.69]), chronic liver disease (3.52 [1.73–7.17]) and migraine (1.81 [1.2–2.74]).

In *2014*, the 15–34 age group had significantly lower odds of having cancer (0.1 [0.02–0.45]) compared to those who are 65 and above. Employed respondents were exposed to cancer with significantly lower odds (0.34 [0.16–0.74]) than the unemployed responder. Urban residents had higher odds of being cancer patients compared to rural areas (1.85 [1.08–3.16]). Participants in the highest income quantile showed a significant association with a higher occurrence of cancer (2.88 [1.34–6.19]). Bad self-reported health was associated with increased prevalence of cancer cases (5.52 [3.23–9.45]) while good self-reported health decreased the prevalence (0.27 [0.11–0.62]). Arrhythmia occurred with higher odds among cancer patients (1.88 [1.1–3.24]).

In *2019*, the 15–34 age group was associated with lower odds of cancer (0.16 [0.04–0.68]). Higher education was closely associated with higher odds (1.99 [1.04–3.83]). Residents of the Northern Great Plains had lower odds of cancer prevalence (0.43 [0.19–0.97]). Good self-perceived health showed lower odds of a being cancer patient (0.31 [0.14–0.68]) conversely bad self-perceived health reported higher (2.23 [1.26–3.95]). Among the chronic conditions, peptic ulcer was associated with higher odds of cancer (3.2 [1.58–6.47]), in contrast, migraine was associated with lower odds (0.3 [0.12–0.75]).

### 3.4 Results of multiple logistic regression analysis on the pooled data

The odds ratio of having cancer in 2014 and 2019 have decreased compared to 2009 as per results in Table 3, with odds ratios (OR) of 0.57 (CI: 0.43–0.76) and 0.59 (CI: 0.44–0.79), respectively, both instances are statistically significant with p < 0.001. Both the youngest (15–34) (0.23 [0.13–0.43]) and the middle-aged (35–64) (0.68 [0.51–0.92]) groups demonstrated significantly lower odds of having cancer compared to the oldest age group (65+), indicating that the odds of having cancer increase with age. Higher education levels were associated with a significantly higher odds of having cancer. In the pooled sample we observed an increasing trend in the odds ratios with higher levels of education: high school (1.61 [1.22–2.13]), university (2.26 [1.59–3.21]). Being employed contribute to lower odds of having cancer (0.64 [0.45–0.92]) in pooled sample. Urban residents have higher odds compared to the rural residents in the pooled sample (1.44 [1.08–1.9]). Reporting bad health was strongly associated with higher odds (3.07 [2.29–4.12]) of having cancer, while reporting good health was associated with decreased odds (0.37 [0.25–0.55]) in the pooled sample. Among the chronic conditions peptic ulcer (1.83 [1.31–2.54]) and chronic liver disease (2.5 [1.4–4.47]) showed significant association.

**Table 3:** Weighted multiple logistic models of factors affecting cancer prevalence in 2009, 2014, 2019 and pooled datasets.

Characteristics		2009		2014		2019		Pooled dataset	
		Adjusted OR (95% CI)	P value	Adjusted OR (95% CI)	P value	Adjusted OR (95% CI)	P value	Adjusted OR (95% CI)	P value
**Year**	2009								
	2014							**0.57 [0.43–0.76]**	**<0.001**
	2019							**0.59 [0.44–0.79]**	**<0.001**
**Gender**	Male								
	Female	1.02 [0.68–1.54]	0.912	1.27 [0.8–2.03]	0.311	1.2 [0.72–1.99]	0.489	1.12 [0.86–1.46]	0.398
**Age groups**	65+								
	15–34	**0.26 [0.12–0.58]**	**0.001**	**0.1 [0.02–0.45]**	**0.003**	**0.16 [0.04–0.68]**	**0.014**	**0.23 [0.13–0.43]**	**<0.001**
	35–64	**0.54 [0.34–0.85]**	**0.008**	0.83 [0.5–1.4]	0.491	0.81 [0.41–1.63]	0.562	**0.68 [0.51–0.92]**	**0.013**
**Educational levels**	Lower than high school								
	High school	**1.81 [1.18–2.8]**	**0.007**	1.54 [0.89–2.66]	0.12	1.51 [0.94–2.42]	0.091	**1.61 [1.22–2.13]**	**0.001**
	University	**2.6 [1.49–4.55]**	**0.001**	1.92 [0.99–3.73]	0.053	**1.99 [1.04–3.83]**	**0.039**	**2.26 [1.59–3.21]**	**<0.001**
**Employment**	Unemployed								
	Employed	0.71 [0.43–1.17]	0.174	**0.34 [0.16–0.74]**	**0.006**	0.84 [0.4–1.77]	0.647	**0.64 [0.45–0.92]**	**0.014**
**Residence area**	Rural								
	Urban	1.34 [0.88–2.06]	0.176	**1.85 [1.08–3.16]**	**0.026**	1.51 [0.85–2.68]	0.162	**1.44 [1.08–1.9]**	**0.012**
**Region**	Central-Hungary								
	Southern Great Plain	1.48 [0.85–2.57]	0.163	0.74 [0.35–1.59]	0.446	0.74 [0.35–1.56]	0.423	1.07 [0.73–1.55]	0.741
	Southern Transdanubia	1.68 [0.91–3.1]	0.098	1.55 [0.73–3.29]	0.258	1.06 [0.52–2.16]	0.864	1.48 [0.99–2.21]	0.053
	Northern Great Plain	0.79 [0.43–1.44]	0.435	0.89 [0.43–1.87]	0.765	**0.43 [0.19–0.97]**	**0.042**	0.72 [0.48–1.07]	0.102
	Central Transdanubia	1.76 [0.96–3.21]	0.066	1.4 [0.72–2.72]	0.322	0.83 [0.39–1.79]	0.635	1.4 [0.95–2.06]	0.089
	Northern Hungary	0.89 [0.47–1.68]	0.711	1.22 [0.59–2.55]	0.588	1.01 [0.46–2.22]	0.975	0.93 [0.62–1.4]	0.736
	Western Transdanubia	0.93 [0.46–1.87]	0.834	0.79 [0.33–1.85]	0.583	1.22 [0.58–2.56]	0.592	0.99 [0.64–1.53]	0.964
**Income quintiles**	First								
	Second	0.58 [0.34–1.02]	0.059	1.26 [0.69–2.31]	0.447	1.6 [0.8–3.21]	0.181	1.01 [0.72–1.42]	0.948
	Third	0.58 [0.33–1.01]	0.053	**2.07 [1.11–3.85]**	**0.021**	1.37 [0.65–2.88]	0.411	1.02 [0.71–1.45]	0.926
	Fourth	0.64 [0.38–1.09]	0.099	1.48 [0.65–3.37]	0.349	0.62 [0.28–1.35]	0.23	0.79 [0.54–1.15]	0.213
	Fifth	**0.47 [0.25–0.88]**	**0.019**	**2.88 [1.34–6.19]**	**0.007**	1.18 [0.47–2.97]	0.721	0.86 [0.57–1.31]	0.482
**BMI**	Overweight/Obese								
	Normal	1.09 [0.75–1.59]	0.641	1.05 [0.68–1.64]	0.816	1.08 [0.67–1.73]	0.745	1.08 [0.84–1.37]	0.557
**Smoking**	Smoker								
	Non-smoker	1.06 [0.7–1.6]	0.774	0.91 [0.54–1.54]	0.724	1.1 [0.66–1.83]	0.726	1.01 [0.77–1.33]	0.951
**Alcohol use**	Drinker								
	Non-drinker	1.32 [0.89–1.95]	0.163	1.2 [0.75–1.92]	0.456	1.34 [0.84–2.13]	0.22	1.29 [1–1.66]	0.048
**Self-perceived health**	Average								
	Good	**0.49 [0.28–0.85]**	**0.011**	**0.27 [0.11–0.62]**	**0.002**	**0.31 [0.14–0.68]**	**0.004**	**0.37 [0.25–0.55]**	**<0.001**
	Bad	**2.92 [1.87–4.58]**	**<0.001**	**5.52 [3.23–9.45]**	**<0.001**	**2.23 [1.26–3.95]**	**0.006**	**3.07 [2.29–4.12]**	**<0.001**
**Asthma**	No								
	Yes	0.75 [0.4–1.4]	0.361	1 [0.45–2.23]	0.998	0.93 [0.41–2.1]	0.853	0.81 [0.53–1.23]	0.328
**Bronchitis**	No								
	Yes	1.57 [0.92–2.65]	0.096	0.83 [0.36–1.9]	0.656	1.01 [0.42–2.39]	0.988	1.24 [0.84–1.82]	0.28
**AMI**	No								
	Yes	0.73 [0.36–1.46]	0.369	0.68 [0.29–1.57]	0.367	0.75 [0.26–2.18]	0.6	0.74 [0.46–1.18]	0.21
**CAD**	No								
	Yes	0.78 [0.44–1.38]	0.393	1.05 [0.54–2.04]	0.885	0.5 [0.19–1.37]	0.179	0.81 [0.56–1.19]	0.28
**Hypertension**	No								
	Yes	1.04 [0.7–1.54]	0.845	0.7 [0.41–1.2]	0.195	1.11 [0.68–1.82]	0.666	0.96 [0.74–1.25]	0.758
**Stroke**	No								
	Yes	**0.42 [0.18–0.99]**	**0.048**	1.75 [0.8–3.82]	0.158	1.6 [0.71–3.62]	0.254	0.92 [0.57–1.47]	0.714
**Chronic back pain**	No								
	Yes	**0.65 [0.43–0.99]**	**0.047**	0.87 [0.52–1.46]	0.6	0.93 [0.58–1.51]	0.776	0.78 [0.6–1.01]	0.061
**Chronic neck pain**	No								
	Yes	1 [0.62–1.61]	0.988	1.07 [0.59–1.92]	0.827	1.27 [0.72–2.23]	0.404	1.04 [0.77–1.42]	0.785
**Diabetes**	No								
	Yes	0.67 [0.39–1.15]	0.146	0.88 [0.47–1.65]	0.684	1.15 [0.6–2.23]	0.668	0.81 [0.57–1.14]	0.225
**Peptic ulcer**	No								
	Yes	**1.74 [1.13–2.69]**	**0.012**	1.9 [0.92–3.91]	0.082	**3.2 [1.58–6.47]**	**0.001**	**1.83 [1.31–2.54]**	**<0.001**
**Chronic liver disease**	No								
	Yes	**3.52 [1.73–7.17]**	**0.001**	2.41 [0.59–9.87]	0.22	0.45 [0.05–3.68]	0.455	**2.5 [1.4–4.47]**	**0.002**
**Migraine**	No								
	Yes	**1.81 [1.2–2.74]**	**0.005**	0.82 [0.4–1.69]	0.599	**0.3 [0.12–0.75]**	**0.01**	1.22 [0.9–1.67]	0.205
	Yes	1.29 [0.78–2.13]	0.315	0.68 [0.31–1.49]	0.334	1.14 [0.46–2.8]	0.781	1.1 [0.76–1.6]	0.601
**Mental illness**	No								
	Yes	1.03 [0.53–2.02]	0.931	0.73 [0.29–1.83]	0.5	0.78 [0.39–1.54]	0.475	0.87 [0.57–1.31]	0.495
**Hypercholesterolaemia**	No								
	Yes	1.58 [1–2.5]	0.05	0.61 [0.34–1.1]	0.098	0.8 [0.44–1.43]	0.45	1.07 [0.79–1.45]	0.674
**Arrhythmia**	No								
	Yes	1.09 [0.69–1.71]	0.708	**1.88 [1.1–3.24]**	**0.022**	1 [0.56–1.79]	0.996	1.23 [0.91–1.66]	0.17

Bold values indicate statistical significance, p<0.05. Adjusted ORs account for other variables within the model.

## 4 Discussion

In our study, we aimed to assess the burden of cancer on the Hungarian population by identifying socio-demographic, lifestyle factors, and co-morbidities potentially associated with the disease. The following interpretations are primarily based on findings from the weighted multiple logistic regression models, which account for potential confounders and provide adjusted associations. According to our research, significantly lower odds were detected for cancer in 2014 compared to 2009, followed by a statistically steady cancer prevalence in 2019. Further drastic decrease would be essential, given that cancer mortality in Hungary remains the highest in Europe [6].

Although certain types of cancer e.g. leukemias and lymphomas, brain and central nervous system tumors, sarcomas of bone are most frequently diagnosed among children and adolescents [17,18], pediatric cancer is a relatively rare disease [19]. There is sufficient evidence that the overall incidence of cancer increases consistently with age [18]. This phenomenon can be attributed to the fact that the number of somatic mutations increases exponentially as people age, due to random errors in DNA replication, endogenous factors (e.g. genetic susceptibility, hormones, inflammations, growth factors etc.) as well as environmental exposures (e.g. ionization radiations, chemical carcinogens, unhealthy lifestyle habits etc.) [20,21]. Meanwhile, DNA repair mechanisms – including mismatch, base excision, nucleotide excision and double-strand break repairs – become less efficient and more error-prone during the aging process [22]. In addition to the accumulation of mutations, the immune functions decline as well in old versus young, known as immunosenescence, losing the potential to eliminate malignant cells [23]. In fact, these changes make age the major risk factor for cancer [18]. Lung, prostate, colorectal, stomach and liver cancers were reported to be the most frequent in adult men, while breast, colorectal, lung, cervical and thyroid cancers were predominant in adult women [1]. Analysing three different age groups (15–34, 35–64 and 65+) in association with the total number of cancer cases in all the examined years (2009, 2014 and 2019), we observed that the odds for cancer in both younger (15–34) and middle age groups (35–64) were significantly lower compared to the population over 65, which confirms the previous studies’ findings: individuals over 65 have the highest chance of developing tumors, which is followed by the middle age group and the lowest risk can be linked to the youngest age group in the population. This trend showed consistent significance for the youngest age group in all examined three years. In the middle age group, significantly lower odds were detected only in 2009 compared to the 65+ age group, but the tendency of growing numbers of cancer cases as a function of age is clearly visible.

Beyond the natural ageing processes, unhealthy diet patterns, obesity, exposure to tobacco, alcohol consumption and physical inactivity have significant impacts on the development of cancer as well [24,25]. Smoking is associated with lung, colorectal, stomach and liver cancers most typically, overweight/obesity and sedentary lifestyle can contribute to breast and colorectal cancers [26], while moderate to heavy alcohol intake can increase the risk of head and neck, esophaegal, liver, breast and colorectal cancers [27]. Furthermore, infections can cause liver, stomach, cervical [26] and head and neck cancers [28].

Higher education can increase the ability to attain or comprehend health-related information in general, which is necessary to develop health-promoting attitudes. Irala-Estévez et al. [29] found that higher levels of education were linked to higher consumption of fruits and vegetables in European countries. Puka et al. [30] demonstrated that unhealthy factors such as smoking, obesity and physical inactivity were more frequent among participants with lower levels of education compared to those of higher degrees. Other studies revealed unequal distribution between cancer types and educational attainment: mid- and high-educational groups had higher incidences of breast [12,31,32], prostate cancer [32,33] and melanoma [32] while the low-educational groups had higher incidence rates for lung [12,32], stomach, nasopharyngeal, and esophageal cancers [12]. Colorectal cancer was reported to be associated to both lower [34] and higher [12] educational levels. Al-Rammahy et al. [32] detected an even distribution of both colon and rectal cancer incidence across the educational groups. Analysing the total number of cases in 2009, 2014 and 2019 in three differently educated groups (lower than high school, high school, university), we detected significantly higher odds for both middle and high educational levels compared to the participants with education lower than high school. Interestingly, the diagnosed cancer cases were the highest in the highest educational category, more, than twofold. Similar odds ratios were found in each year, with significant differences in 2009 for the high school and university groups, and in 2019 for the university group. These observations may confirm the multifactorial complexity of cancer development [20], i.e. there may be additional factors, which are unknown within the scope of the current survey, while these results may also be explained by the more health-conscious and protective mind-set of people with higher education, who are more likely to participate in screening programmes [15] and thus more cancer cases can be discovered in this population, even in early state that can contribute to a more positive outcome of the disease [35].

Besides educational attainment, area of residence is another strong determinant of lifestyle behaviours. Due to the different lifestyle habits and environmental exposures of rural and urban areas, risk and protective factors may differ in different geographic regions. Thus, different types of cancer may also vary at local levels [36,37]. In our data, we found significantly higher odds for cancer among urban residents compared to rural dwellers in the pooled sample. The most significant odds ratio was observed in 2014. Air pollutants as carcinogenic agents are typically linked to urban areas with an increased risk of lung cancer [38] as a consequence of industrialization and economic development. In 2009, 2014 and in 2019 we detected the highest odds for cancer in Central Transdanubia, Southern Transdanubia and Western Transdanubia regions of Hungary, respectively, compared to Central Hungary, which is the capital and its surrounding area, economic, commercial, financial, administrative and cultural centre of Hungary, and we detected the lowest odds for cancer in either the Northern or Southern Great Plains in each year. Although the differences were not significant statistically, with the exception of the significantly lower odds ratio for the Northern Great Plain in 2019, these results might be in accordance with the Hungarian territorial specificities, i.e. Transdanubian regions – in particular Central and Western Transdanubia – are relatively developed and industrialised regions, while in the Northern or Southern Great Plains agriculture is the main profile [39].

Analysing the associations between employment and cancer in the pooled sample, we detected significantly lower odds for cancer among employed individuals compared to unemployed participants. Prolonged unemployment is reported to be linked to both mental and physical health problems including cancer [40], while many studies highlight the possible consequence of cancer: the risk of job loss among survivals [41,42]. It is highly plausible that this vulnerability is reflected by our data, which was the most prominent in 2014, potentially and apparently resulting drastic impact on the household incomes that year: the odds for the disease was almost three times higher among those, who rated their household incomes as the worst (fifth quintiles) compared to those with very good incomes (first quintiles).

Self-perceived health is an epidemiological indicator of general health as well as predictor of survival time and mortality caused by cancer [43]. The findings of our research are in line with previous studies [44] suggesting a very strong inverse correlation in each year between self-perceived health and cancer: the odds were significantly lower and markedly higher for developing cancer among those participants, who rated their health as good and bad, respectively compared to those respondents, who estimated their health as average, i.e. decreasing self-rated health is associated with increasing risk of cancer.

In terms of co-morbidities, positive associations were found for cancer in case of peptic ulcer and chronic liver disease with significantly higher odds in the pooled analyses. Based on our data, peptic ulcer patients were affected regarding the risk of cancer to the largest extent in 2009 and in 2019, whereas individuals with chronic liver disease might have experienced the most significant effect in 2009, most likely due to the fact that both of the health issues are reported to be linked to cancer. People with a history of peptic ulcers are at increased risk of developing stomach cancer [45,46]. Most commonly, *Helicobacter pylori* infection and the use of non-steroidal anti-inflammatory drugs (NSAIDs) are responsible for the development of peptic ulcers, other risk factors include smoking, alcohol consumption and stress [47]. Chronic liver disease (CLD) can result in complications such as cirrhosis and the most frequent type of liver cancer, hepatocellular carcinoma. CLD can be caused by viral infections (Hepatitis B and C), obesity, metabolic syndrome (non-alcoholic fatty liver disease) and its relationship with excessive use of alcohol is also well established (alcohol-related liver disease) [48]. Interestingly, no significant associations were found with body mass index (BMI), smoking and alcohol consumption with cancer in the examined population, however, it may be biased due to the self-response nature of the survey.

In addition, we detected significantly higher odds for cancer among patients with migraine in 2009, and among individuals diagnosed with arrhythmia in 2014 in comparison to those without any of these health conditions. Headaches [49] including migraine can be associated to different types of head and neck cancers due to the consequence of a complex interaction between cancer cells, their microenvironment and the overlapping systemic physiological processes during carcinogenesis [50] and they can occur as complications of surgical, chemotherapy and/or radiotherapy treatments [49]. Besides, certain anticancer agents (e.g. anthracyclines and antimetabolites) and immunotherapies carry a risk of cardiotoxicity, and therefore an increased risk of cardiac arrhythmias also can be observed in oncology patients [51]. We detected significantly lower odds among patients with chronic back pain and stroke in 2009, contrary to expectations, since many cancers – mainly breast, prostate, lung and kidney – can metastasise into bones including the spine causing chronic pain [52], furthermore, these cancer types as well as pancreatic, colorectal cancers, as the most common types, have been shown to be associated with ischaemic or haemorrhagic stroke [53] via hypercoagulability, non-bacterial thrombotic endocarditis, direct tumor compression of blood vessels [54,55], therefore further analysis is needed to reveal all the possibly interacting factors resulting our findings.

## 5 Strengths and limitations

The data were collected using the standardized European Health Interview Survey questionnaire, supervised by Eurostat, and provided by the Central Statistical Office of Hungary for analysis. The sample is representative of the Hungarian population, enhancing its external validity and generalizability. To our knowledge, results from a similarly large and representative sample have not yet been published for the Hungarian population on this topic.

Our research has certain limitations inherent to surveys with a cross-sectional design. The self-reported nature of the data may introduce response bias, and as a cross-sectional study, it captures prevalence rather than incidence or mortality, limiting causal and temporal insights. Survival bias is also present, as data include only those alive at survey time, leading to a higher representation of female cancer survivors due to longer survival rates for certain cancers. Additionally, the absence of key clinical markers limits differentiation by disease severity, and the lack of cancer type stratification and regional detail, such as separating Pest county from Budapest, restricts specific subgroup analyses.

## 6 Conclusions

The current study confirms that socio-demographic characteristics can influence disease incidence, and the ability to work, employment, financial resources, hence, presumably even the access to further cancer care. We have shown existing co-morbidities either as causes or consequences of the disease, which can significantly affect quality of life and determine life expectancy, however, further analysis and research are needed to explore more specific interactions and the exact causal relationships behind these observations.
